# Isolation of Lactic Acid Bacteria from Naturally Ensiled *Rosa roxburghii* Tratt Pomace and Evaluation of Their Ensiling Potential and Antioxidant Properties

**DOI:** 10.3390/foods14081329

**Published:** 2025-04-11

**Authors:** Xiong Pan, Yafei Zhang, Ningbo Yue, Ke Yu, Lang Zhou, Lijuan Ge, Faju Chen, Juan Yang, Qiji Li, Tingfei Deng, Xiaosheng Yang

**Affiliations:** 1 State Key Laboratory of Discovery and Utilization of Functional Components in Traditional Chinese Medicine, School of Pharmaceutical Sciences, Guizhou Medical University, Guiyang 550014, China16683864072@163.com (Y.Z.); yangxz2002@126.com (J.Y.); dengtingfie@sina.com (T.D.); 2 Natural Products Research Center of Guizhou Province, Guiyang 550014, China; 3 Qiannan Academy of Agricultural Sciences, Duyun 558000, China

**Keywords:** lactic acid bacteria, *Rosa roxburghii Tratt* pomace, isolation, identification, antioxidant properties, preservation potential

## Abstract

This study isolated five acid-producing strains (XQ_1_ and YZ_1_–YZ_4_) from naturally fermented pomace of *Rosa roxburghii* Tratt (RRT) in Guizhou’s karst region. Genetic and phenotypic analyses identified XQ_1_, YZ_2_, and YZ_4_ as *Lactobacillus plantarum* (*L. plantarum*), YZ_3_ as *Weissella cibaria*, and YZ_1_ as *Bacillus licheniformis*. A comparative evaluation with commercial strain AC revealed that XQ_1_, YZ_2_, and YZ_4_ exhibited superior acidification (reaching the stationary phase at 40 h) and tolerance to acidic conditions (pH 3.0), ethanol (6% *v*/*v*), bile salts (0.3%), and osmotic stress (6.5% NaCl), along with broad-spectrum antimicrobial activity against *Bacillus subtilis*, *Staphylococcus aureus*, *Escherichia coli*, *Shigella dysenteriae*, and *Pseudomonas aeruginosa*. Their cell-free supernatants (CFSs) showed comparable superoxide dismutase activity and total antioxidant capacity (2.54–2.66 FeSO_4_·7H_2_O eq mmol/L) to AC (2.68), with DPPH radical scavenging exceeding 50%. YZ_3_ displayed weaker acid production, tolerance, and limited antimicrobial effects. Safety assessments confirmed non-hemolytic activity and antibiotic susceptibility. In conclusion, the *L. plantarum* strains XQ_1_, YZ_2_, and YZ_4_ demonstrated strong ensiling potential and remarkable probiotic properties, establishing them as promising indigenous microbial resources for the preservation of RRT pomace and other food products.

## 1. Introduction

*Rosa roxburghii* Tratt (RRT), an edible and medicinal plant, is classified under the Rosa genus within the Rosaceae family [1]. Renowned for its rich nutritional and functional components, RRT contains an abundance of polysaccharides, amino acids, vitamin C, superoxide dismutase (SOD), flavonoids, triterpenoids, and phenolic compounds [2]. These bioactive constituents endow RRT with significant nutritional and medicinal properties, including antioxidant and antitumor activities, radiation protection, atherosclerosis prevention, and the inhibition of apoptosis [3]. The primary distribution of RRT is in southwestern China, with Guizhou Province serving as its main cultivation region. Characterized by karst topography, Guizhou experiences highly variable microclimatic conditions, with notable temperature fluctuations between day and night, a predominantly cool environment, and elevated humidity levels. Such distinctive climatic factors contribute to the superior quality of RRT produced in this region. Consequently, the cultivation of RRT in Guizhou has undergone substantial expansion, with an annual planting area exceeding 50,000 hectares, driven by its remarkable nutritional and medicinal benefits [4]. The predominant processing approach for RRT involves juicing to produce RRT juice products. This production primarily occurs from mid-August to late September, resulting in a highly concentrated processing period. Moreover, the by-product generated from juice extraction, RRT pomace (abbreviated as RRTP), is subject to a short storage period, which poses challenges for large-scale drying and preservation. Due to these constraints, a considerable quantity of RRTP (the residual material following juice extraction) is discarded, highlighting the necessity for the development of an effective treatment and disposal strategy.

Most research findings have indicated that RRTP retains a substantial amount of nutritional and functional constituents, suggesting its potential application as a functional food ingredient or in other product formulations. Ensiling has been recognized as an effective approach for the long-term preservation of pomace [5,6], preventing spoilage while garnering increasing attention. Lactic acid bacteria (LAB) play a crucial role in enhancing silage quality and safety through multiple mechanisms during the ensiling process. Firstly, LAB synthesize substantial quantities of lactic acid and other beneficial organic acids through carbohydrate fermentation, rapidly acidifying the environment to pH levels (typically < 4.5) that effectively inhibit the proliferation of spoilage microorganisms [7]. Secondly, the metabolic activities of LAB lead to the production of various aromatic compounds, such as alcohols and esters, which improve the organoleptic properties of the silage and enhance its palatability for silage [8]. Thirdly, LAB can modulate microbial community dynamics and functional shifts during the ensiling process [9]. Furthermore, LAB exhibit antioxidant capabilities through the biosynthesis of compounds that effectively neutralize free radicals. This antioxidant activity mitigates the oxidative degradation of vital nutrients such as vitamins and unsaturated fatty acids, thereby preserving the nutritional integrity of the feed throughout storage [10]. Throughout the ensiling process, the availability and type of LAB serve as critical determinants influencing preservation efficacy. LAB are capable of generating natural antimicrobial and antioxidant compounds, including organic acids, hydrogen peroxide, carbon dioxide, ethanol, diacetyl, γ-aminobutyric acid, bacteriocins, and bacteriocin-like inhibitory substances, thereby exerting a preservative effect [11].

Compared to exogenous LAB, epiphytic LAB associated with plants exhibit superior performance in ensiling applications. The species of raw materials, in turn, exert an influence on the community composition of epiphytic LAB [12]. Furthermore, Guizhou, characterized by its karst landform, possesses a distinctive altitude and climatic conditions. These environmental factors not only contribute to the exceptional quality of Guizhou RRT but also foster rich microbial diversity. Consequently, the isolation of LAB from RRTP produced in Guizhou can be utilized as a starter culture to enhance RRTP biopreservation, thereby increasing its added value. Additionally, it serves as a promising source for identifying probiotic strains with superior functional potential.

Guizhou Province, distinguished by its karst landforms, harbors a diverse microbial community. The objective of this study was to isolate beneficial LAB from naturally ensiled RRTP in Guizhou Province and to conduct a systematic assessment of their ensiling potential, including growth dynamics, acid production capacity, antimicrobial properties, and tolerance. Additionally, their antioxidant activity and safety profile were evaluated with the aim of providing indigenous bacterial resources for the future preservation of RRTP or other food products.

## 2. Materials and Methods

### 2.1. Samples

Fresh RRTP: (Sinopharm Group Health Industry Development Co., Ltd., Guizhou, China). Indicator strains (including *Escherichia coli*, *Staphylococcus aureus*, *Bacillus subtilis*, *Pseudomonas aeruginosa*, *Shigella dysenteriae*, and *Mycobacterium smegmatis*): (Guizhou Natural Products Research Center, Guizhou, China). The commercial *L. plantarum* (preservation number ACCC11016, named AC in this study): (Agricultural Culture Collection, Beijing, China). AC demonstrates high efficacy in silage preservation and is widely employed as a microbial inoculant.

### 2.2. Spontaneous RRTP Silage Preparation and Sampling

Fresh RRTP (800 g) was placed into polyethylene plastic bags (200 mm × 300 mm), vacuum-sealed using a vacuum extractor, and subsequently stored at room temperature (25 ± 2 °C) for one month. The resulting RRTP silage was utilized for microbiological analysis aimed at LAB isolation.

### 2.3. Isolation of Acid-Producing Strains from RRTP

Strains exhibiting acid-producing capability were selected by incorporating 1% calcium carbonate into a conventional de Man, Rogosa, and Sharpe (MRS) medium [9]. A total of 2 g of naturally ensiled RRTP was transferred into a sterile bottle containing 200 mL of sterile water and subsequently incubated in a shaking incubator at 28 °C for 24 h. Following incubation, 30 μL of the resulting suspension was uniformly spread onto MRS agar plates supplemented with 1% calcium carbonate. Single colonies that exhibited calcium dissolution zones were isolated and further purified using the continuous streak plate technique on MRS agar plates.

### 2.4. Phenotype Characterization of the Isolated RRTP LAB Strains

The physiological and biochemical characterization of acid-producing isolates was performed through a series of assays, including carbohydrate fermentation, acid production, glucose gas production, catalase activity, motility assessment, and starch hydrolysis [13]. In the carbohydrate fermentation test, 2% of various sugars was incorporated into the PY basic liquid medium, followed by inoculation with purified strains and the addition of a bromocresol purple indicator. After 24 h of incubation, a yellow color change in the medium indicated acid production [14]. For glucose gas production analysis, a PY basic medium containing 2% glucose was drawn into Durham’s fermentation tubes using a syringe, ensuring the removal of air bubbles. The tubes were inverted along the inner wall of the test tube and fully submerged in the respective medium. Following inoculation with purified strains and 24 h of incubation, the presence of floating Durham’s fermentation tubes and bubble formation signified gas production. Gram staining was conducted utilizing a Gram stain kit. Catalase activity was assessed by applying a drop of 3% hydrogen peroxide onto a slide, followed by streaking purified strains onto the surface. The formation of bubbles indicated positive catalase activity. Motility was examined by stab-inoculating purified strains into a semi-solid MRS medium. For starch hydrolysis assessment, purified strains were cultivated for 24 h in an MRS medium supplemented with 0.5% soluble starch to prepare bacterial suspensions. A drop of the bacterial suspension was placed onto a slide, followed by the addition of one drop of Lugol’s iodine solution. A blue-black coloration signified the absence of starch hydrolysis, whereas the presence of clear zones indicated a positive starch hydrolysis reaction.

### 2.5. Genotypic Identification of Presumptive LAB Isolates

The genetic identification of the isolated strains was conducted by Sangon Biotech (Shanghai, China) Co., Ltd., following the methodology outlined by Singh [15]. Genomic DNA extraction was carried out using the magnetic bead method per the kit’s instructions. Sample plates, magnetic bead plates, washing plates, and elution plates were prepared and positioned in designated locations within the extraction instrument, after which the bacterial extraction program was executed until completion. For bacterial 16S rRNA gene amplification, the universal primers 27F and 1492R were employed. The reaction system (21 μL) comprised 21 μL polymerase chain reaction (PCR) mix, 2 μL template DNA, and 1 μL each of Primer F (5p) and Primer R (5p). The PCR conditions were set as follows: an initial pre-denaturation at 96 °C for 5 min, followed by 35 cycles of denaturation at 96 °C for 30 s, annealing at 56 °C for 1 min, and extension at 72 °C for 1 min, concluding with a final extension at 72 °C for 5 min. For PCR product analysis and purification, 3 μL of the amplified PCR product was subjected to 1.0% agarose gel electrophoresis to evaluate band characteristics. Purification of the PCR products was performed following the standard magnetic bead purification protocol. Sequence analysis was conducted using NCBI-BLAST, and a phylogenetic tree was generated utilizing the neighbor-joining method in MEGA11.0.

### 2.6. Evaluation of the Ensiling Potential of Isolated LAB

#### 2.6.1. Determination of Growth Curve and Acid-Producing Ability

Following the modified methodology of Jain et al. [14], 20 mL of activated bacterial culture (OD600 = 1) was introduced into 200 mL of MRS broth. The culture was incubated at 37 °C with agitation at 160 r/min, and samples were collected at 5 h intervals until the stationary phase to assess acid production and bacterial growth capacity. For the evaluation of growth capacity, 5 mL of culture broth was extracted, and the OD600 value was recorded using the 0 h culture medium as the reference control. Acid production was assessed by determining the pH of the culture broth with a digital pH meter following the measurement of growth capacity.

#### 2.6.2. Antibacterial Activity of Isolated LAB

The LAB cell-free supernatant (CFS) was prepared in accordance with the procedure outlined in Section 2.7.1. The inhibitory activity of the isolated strains’ CFSs against indicator strains was assessed using the agar diffusion method [16]. Indicator strains (1%, *v*/*v*) were introduced into a nutrient agar medium and allowed to solidify. Wells were subsequently created in the agar, into which 100 μL of supernatant was dispensed. The plates were incubated at 37 °C for 24 h, after which the diameters of the inhibition zones were measured. The indicator strains tested included *Bacillus subtilis*, *Staphylococcus aureus*, *Escherichia coli*, *Shigella dysenteriae*, and *Pseudomonas aeruginosa*.

#### 2.6.3. Ethanol, Acid, Osmotic Pressure, and Bile Salt Tolerance

The OD600 spectrophotometric method, which establishes a positive correlation between bacterial concentration and absorbance at a wavelength of 600 nm, was utilized as a bacterial enumeration technique. The tolerance of the purified isolated strains was assessed using this OD600-based approach [17]. Given that the ensiling process is highly susceptible to external environmental fluctuations and that the proliferation capacity of LAB is influenced by these variations, LAB intended for RRTP preservation should exhibit a degree of tolerance to environmental changes. The tolerance of XQ_1_ was evaluated across four parameters. For each experimental condition, LAB cultures were activated in MRS broth for 40 h before being individually inoculated at 1 μL into corresponding MRS broth containing varying concentrations of specific additives. Ethanol tolerance was examined using ethanol concentrations of 2%, 4%, 6%, 8%, and 10%. Osmotic pressure tolerance was determined with NaCl concentrations of 3%, 4%, 5%, 6%, 7%, and 8%. Bile salt tolerance was analyzed by supplementing the medium with porcine bile salt at concentrations of 0.05%, 0.1%, 0.5%, 1%, and 1.5%. Acid tolerance was tested at pH values of 1.5, 2.5, 3.5, 4.5, and 5.5, with pH adjustments made using 1 mmol/L HCl. Following 24 h of incubation, bacterial growth was quantified by measuring absorbance at 600 nm [18].

### 2.7. Antioxidant Activity

#### 2.7.1. Preparation of Cell-Free Supernatant (CFS), Intact Cells (ICs), and Cell-Free Extract (CFE)

The preparation of the CFS, ICs, and CFE was adapted from the method outlined by Hongyu Wang [19]. In summary, following bacterial activation in MRS broth, the culture was subjected to centrifugation (10,000× *g*, 20 min, 4 °C) and subsequently filtered using a 0.22 μm membrane filter to obtain the supernatant (CFS). After activation and centrifugation, bacterial cells were resuspended in a phosphate-buffered saline (PBS) solution at an approximate concentration of 1 × 10⁹ CFU/mL, yielding ICs. Additionally, the activated bacterial culture underwent sonication, followed by centrifugation (10,000× *g*, 20 min, 4 °C) to remove cellular debris. The resulting supernatant was then filtered through a 0.22 μm membrane filter to obtain the CFE.

#### 2.7.2. Superoxide Dismutase (SOD) Activity

In accordance with the manufacturer’s protocol (Nanjing Jiancheng), the activity of SOD was quantified utilizing the total superoxide dismutase detection kit based on the WST-1 method [20].

#### 2.7.3. DPPH Free Radical Scavenging Activity

DPPH• is a stable purple free radical that undergoes reduction to 2,2-diphenyl-1-picrylhydrazine (pale yellow) upon reaction with antioxidants [20]. In accordance with the manufacturer’s protocol (Yuanye), the DPPH radical scavenging activity was assessed utilizing a DPPH radical scavenging assay kit. Absorbance was recorded at 517 nm using a microplate reader, and the radical scavenging activity was computed using the following formula:

DPPH scavenging rate (%) = [A_0_ – (A_1_ – A_2_)]/A_0_ × 100%

where A_0_ represents the absorbance of the blank control, A_1_ represents the absorbance of the sample test tube, A_2_ represents the absorbance of the sample control tube, and A_3_ represents the absorbance of the positive control tube.

#### 2.7.4. Ferric Reducing Antioxidant Power (FRAP)

The total antioxidant capacity was assessed utilizing the FRAP assay kit [21]. A standard curve was generated by correlating the final Fe^2+^ concentration (x, mmol/L) with the standard absorbance (y, ΔA standard). The FeSO_4_ standard curve was established, and the antioxidant activity of the samples was expressed in terms of Fe^2+^ equivalency.

### 2.8. Safety Evaluation of Isolated LAB

#### 2.8.1. Antibiotic Sensitivity

The antibiotics utilized in this study comprised ampicillin, vancomycin, gentamicin, kanamycin, streptomycin, erythromycin, clindamycin, tetracycline, and chloramphenicol, selected in accordance with the recommendations provided by the European Food Safety Authority (EFSA) [22]. The antibiotic susceptibility of the isolated strains was evaluated following EFSA’s guidelines for antimicrobial susceptibility testing of bacteria relevant to human and veterinary health [22], with methodological reference to the approach described by Wu et al. [23].

#### 2.8.2. Hemolysis Test

The LAB strains were activated and subsequently inoculated onto Columbia blood agar plates utilizing the streak plate method, with *Staphylococcus aureus* serving as the positive control. Following incubation at 37 °C for 48 h, the hemolytic activity of the strains was examined.

### 2.9. Data Analysis

All experiments were conducted in triplicate, and statistical analysis was performed using SPSS 25.0. Data are presented as means ± SD based on three replicates. A One-way analysis of variance (ANOVA) was employed to determine statistical significance. Graphical representations were created using Origin 2021.

## 3. Results and Discussion

### 3.1. Isolation and Phenotypic Characterization of Presumptive LAB Isolates

**Figure 1:**
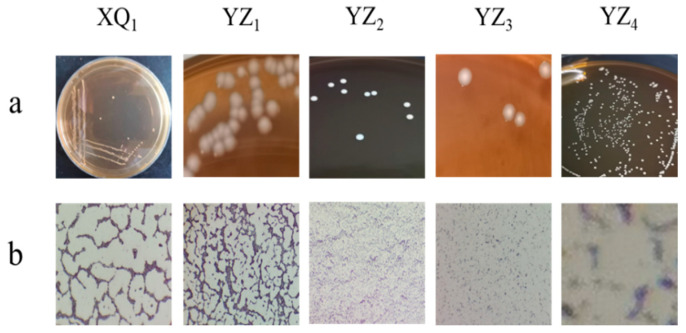
Surface morphology (**a**) and Gram staining microscopic morphology (**b**) of five suspected LAB strains.

Five strains capable of forming calcium-dissolution zones on MRS calcium carbonate medium were successfully isolated from naturally ensiled RRTP. The surface morphology and microscopic Gram staining observations are presented in Figure 1 and Table 1. The colonies of XQ_1_, YZ_2_, and YZ_4_ exhibited a white, circular appearance with a smooth texture, while microscopic analysis confirmed that they were rod-shaped and Gram-positive. YZ_1_ formed circular, raised colonies with a smooth and moist texture, displaying a milky white coloration, with microscopic examination also identifying rod-shaped, Gram-positive bacteria. YZ_3_ developed colonies characterized by bright white centers, serrated edges, and a rough texture, with microscopic analysis revealing rod-shaped, Gram-positive bacteria.

The physiological and biochemical characteristics of the isolated strains are presented in Table 2. Variations in carbohydrate fermentation capabilities were observed among different strains. All strains exhibited acid production and demonstrated the ability to ferment glucose, fructose, maltose, salicin, cellobiose, and esculin. In the catalase assay, YZ_1_ displayed positive catalase activity and motility, whereas the remaining four strains tested negative. Based on the LAB identification criteria established by Amelia et al. [24], YZ_1_ was not preliminarily classified as LAB, while XQ_1_, YZ_2_, YZ_3_, and YZ_4_ were initially identified as LAB strains. Furthermore, the gas-producing strain YZ3 exhibited characteristics of heterolactic fermentation [25].

### 3.2. Genotypic Identification

**Figure 2:**
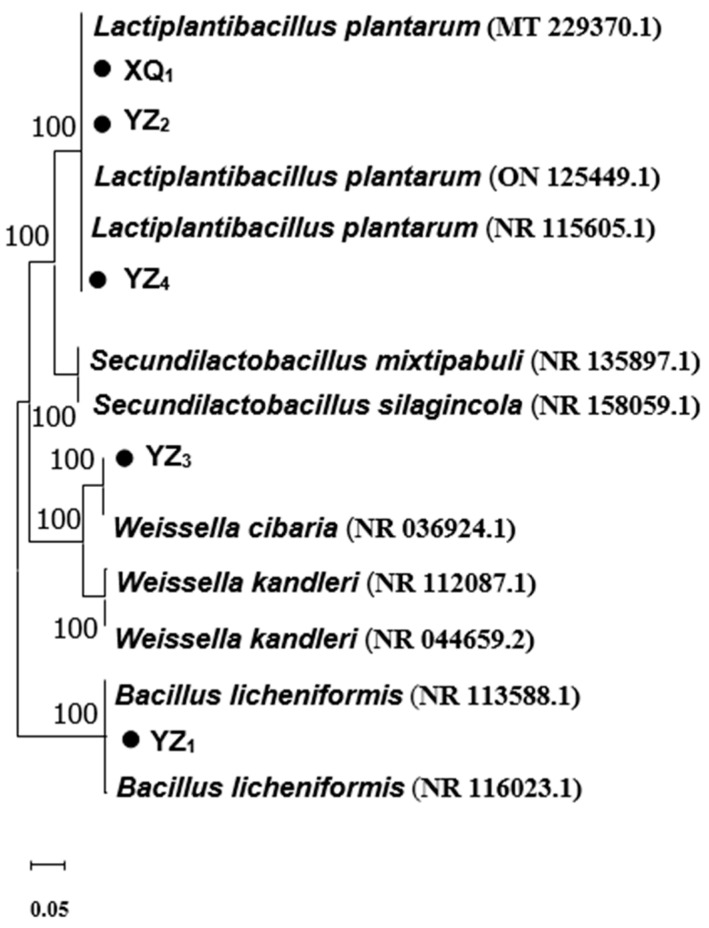
Phylogenetic tree based on 16S rRNA gene sequences of isolated strains. The length of branches represents evolutionary distance, and the numbers on the tree indicate confidence levels.

The sequence similarity of the obtained sequencing results was analyzed using the National Center for Biotechnology Information (NCBI) database through BLAST+2.16.0 (Table 3), and a phylogenetic tree was subsequently constructed (Figure 2). The homology analysis revealed that XQ_1_ exhibited 100% similarity to the *Lactobacillus plantarum* strain (DMR17), YZ_1_ shared 99.9% homology with the *Bacillus licheniformis* strain (BCRC 11702), YZ_2_ displayed 100% similarity to the *Lactiplantibacillus plantarum* strain (HBUAS59638), YZ_3_ demonstrated 99.9% homology with the *Weissella cibaria* strain (II-I-59), and YZ_4_ showed 99.9% similarity to the *Lactiplantibacillus plantarum* strain (JCM 1149). Consequently, XQ_1_, YZ_2_, and YZ_4_ were classified as *Lactobacillus plantarum*, YZ_3_ as *Weissella cibaria*, and YZ_1_ as *Bacillus licheniformis*, aligning with the physiological and biochemical identification results. The sequences exhibiting the highest similarity scores and total scores obtained from BLAST were considered the closest relatives of the isolates. All identified strains displayed greater than 99% similarity with sequences in the NCBI nucleotide database. The phylogenetic tree analysis further validated the identification and classification of the isolates (Figure 2), where each LAB isolate (indicated by triangles) was clustered with type strains from the database. Although Bacillus licheniformis is not categorized as LAB and lacks ensiling potential, previous studies have reported its probiotic properties and industrial relevance [26].

### 3.3. Ensiling Potential

#### 3.3.1. Growth and Acid Production Capacity

**Figure 3:**
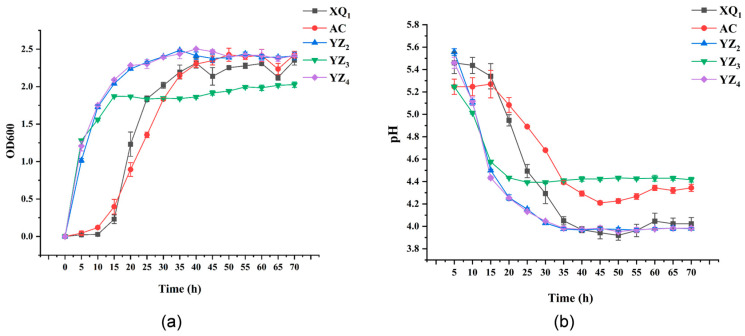
Growth capacity (**a**) and acid production capacity (**b**) of isolated LAB strains and commercial strain AC.

The capacity for acid production and the growth rate are essential parameters for selecting high-quality LAB strains. Rapid proliferation enables LAB to establish dominance within fermented substrates, thereby outcompeting and suppressing the activity of other aerobic bacteria. LAB contribute to the formation of an acidic environment and facilitate the production of substantial quantities of lactic acid due to their strong acidogenic capability, effectively inhibiting the proliferation of pathogenic bacteria [27,28]. An analysis of the growth rates of various LAB strains (Figure 3a) revealed that YZ_2_ and YZ_4_ exhibited the most rapid growth within the first 15 h, whereas AC and XQ_1_ displayed the slowest rates. YZ_2_, YZ_3_, and YZ_4_ entered the logarithmic growth phase following 5 h of cultivation, whereas AC and XQ_1_ reached this phase after 15 h. The transition into the stationary phase occurred at 35 h for AC, XQ_1_, YZ_2_, and YZ_4_, while YZ_3_ reached this phase at 15 h. These variations among LAB strains influenced the timing of their progression into logarithmic and stationary phases [29]. By 40 h, all strains had attained a stable growth phase, fulfilling the criteria for the strong growth capability required for ensiling strains [29]. An evaluation of acid production rates (Figure 3b) indicated that YZ_3_ exhibited stabilization in acid production after 20 h, potentially due to its early entry into the stationary phase (15 h), resulting in a corresponding MRS liquid medium pH of approximately 4.4. AC, XQ_1_, YZ_2_, and YZ_4_ demonstrated stabilized acid production after 40 h, with final pH values of 4.3, 3.9, 4.0, and 4.0, respectively, after 45 h of fermentation. Acid production capacity is a fundamental trait of LAB, as they generate lactic acid and other organic acids through carbohydrate metabolism, leading to a reduction in environmental pH. This acidification not only inhibits the growth of detrimental microorganisms but also influences LAB’s metabolic functions and survival conditions. For example, in food fermentation, organic acids synthesized by LAB serve to suppress spoilage and pathogenic bacteria, thereby extending the shelf life of food products. Additionally, the acidic conditions foster LAB growth, as these bacteria are well adapted to lower pH levels. The disparity in acid production among different strains may be attributed to variations in metabolic pathways and enzyme activity [29]. From a commercial perspective, rapid growth ability can significantly increase the bacterial yield per unit of time, effectively reduce production costs, and enhance the market competitiveness of products. The good acid production capacity can not only regulate the flavor and quality of fermented foods but also create an antibacterial environment by reducing the pH value of the system. Thus, based on the assessment of growth rates and acidogenic capacity, the isolated strains XQ_1_, YZ_2_, and YZ_4_ were identified as potential candidates for RRTP ensiling preservation.

#### 3.3.2. Antibacterial Activity

**Figure 4:**
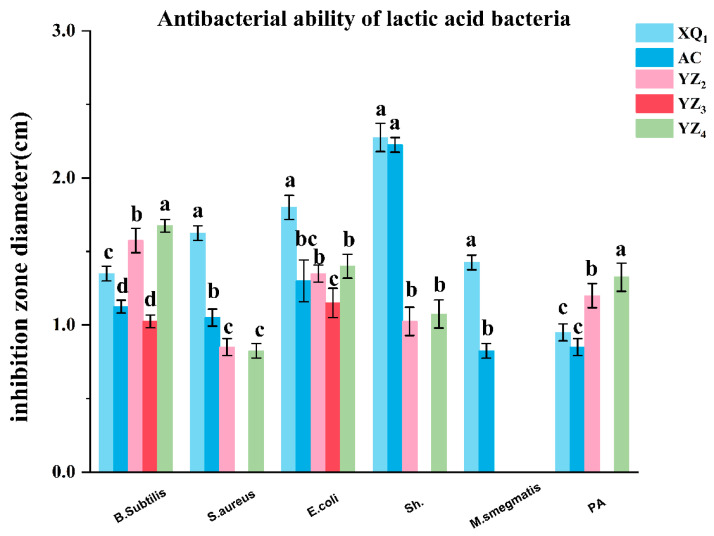
Antibacterial activity of isolated strains. Means annotated with different lowercase letters (a–d) denote significant differences (*p* < 0.05).

Antibacterial activity represents a fundamental characteristic of potential probiotic strains. In this study, *Bacillus subtilis*, *Staphylococcus aureus*, *Escherichia coli*, *Shigella dysenteriae*, and *Pseudomonas aeruginosa* were selected as indicator strains. The inhibition zone diameters of the isolated LAB strains against these pathogenic indicators are depicted in Figure 4. The results indicated that both XQ_1_ and the control strain AC exerted inhibitory effects against all tested indicator strains, with XQ_1_ displaying markedly stronger inhibition than AC against *Bacillus subtilis*, *Staphylococcus aureus*, *Escherichia coli*, and *Shigella dysenteriae*. YZ_2_ and YZ_4_ exhibited comparable antimicrobial properties, effectively inhibiting all indicator strains except *Shigella dysenteriae* and demonstrating markedly greater inhibition against *Bacillus subtilis* and *Pseudomonas aeruginosa* than the AC strain. Conversely, YZ_3_ exhibited only weak inhibitory effects against *Bacillus subtilis* and *Escherichia coli*.

LAB have been extensively recognized as safe and serve an essential function in food fermentation, production, and preservation [30]. Their antimicrobial properties against pathogenic and spoilage microorganisms contribute markedly to inhibiting undesirable microbial fermentation. The isolated strains XQ_1_, YZ_2_, and YZ_4_ in this study exhibited inhibitory effects against indicator pathogenic bacteria, including *Staphylococcus aureus*, *Escherichia coli*, *Shigella dysenteriae*, and *Pseudomonas aeruginosa*, suggesting their potential as candidate strains for RRTP ensiling preservation. Previous studies have reported that the antimicrobial activity of most LAB strains primarily originates from their termina fermentation metabolites [31], which include organic acids and bacteriocins [32,33]. The antimicrobial properties of the isolated strains in this study may not only be attributed to the production of organic acids but could also involve other bioactive compounds, necessitating further investigation in future research.

#### 3.3.3. Tolerance

**Figure 5:**
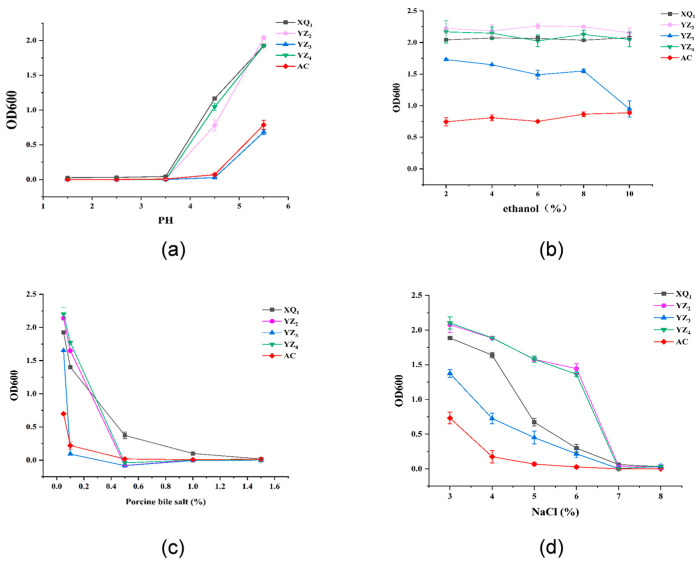
Acid tolerance (**a**), ethanol tolerance (**b**), bile salt tolerance (**c**), and osmotic pressure tolerance (**d**) of isolated LAB strains and commercial strain AC.

Acid, ethanol, bile salt, and osmotic pressure tolerance serve as essential indicators in assessing the probiotic properties of LAB. As illustrated in Figure 5a, strains XQ_1_, YZ_2_, and YZ_4_ exhibited OD600 > 0.5 at pH 4.5, whereas the control strain AC and the isolated strain YZ_3_ exhibited OD600 values close to zero. This suggests that XQ_1_, YZ_2_, and YZ_4_ retained the capability to proliferate under pH 4.5 conditions, in contrast to AC and YZ_3_, which were unable to do so. The pronounced acid tolerance of XQ_1_, YZ_2_, and YZ_4_ would enhance their adaptability to the low-pH conditions encountered during ensiling. With regard to ethanol tolerance (Figure 5b), Sini Kang et al. [34] documented that merely 5 out of 318 isolated bacterial strains were capable of surviving in MRS broth supplemented with 10% (*v*/*v*) ethanol, albeit with reduced growth rates. Interestingly, in the current study, aside from YZ_3_, which exhibited a sharp decline in OD600 at a 10% ethanol concentration, the remaining strains displayed only minor variations, with XQ_1_, YZ_2_, YZ_4_, and AC exhibiting remarkable ethanol tolerance (10%). Bile salt tolerance is a crucial factor in determining the survivability of LAB within the intestinal environment [35]. LAB counteract bile salt stress by activating surface proteins that uphold cell membrane integrity [36]. As depicted in Figure 5c, at a bile salt concentration of 0.1%, YZ_3_ lost its ability to reproduce, while AC exhibited minimal proliferative capacity. However, XQ_1_, YZ_2_, and YZ_4_ maintained significant viability. Even at a 0.3% bile salt concentration, these three strains sustained their activity. Once the bile salt concentration reached 0.5%, only XQ_1_ continued to grow, while all other strains became entirely inactivated. Previous research has established 0.3% as the critical threshold for bile salt tolerance, with strains capable of surviving at this level demonstrating strong resistance [35]. These findings confirm that XQ_1_, YZ_2_, and YZ_4_ exhibit substantial bile salt tolerance, with XQ_1_ performing most effectively, while YZ_3_ remains highly susceptible to bile salts. Regarding osmotic pressure tolerance (Figure 5d), increased NaCl concentrations led to varying degrees of bacterial growth inhibition. At a 5% NaCl concentration, the control strain AC was entirely inactivated, whereas other strains retained their ability to proliferate. At 6% NaCl, XQ_1_ and YZ_3_ exhibited markedly reduced activity, whereas YZ_2_ and YZ_4_ continued to sustain a certain level of growth (OD600 > 1.5). At 7% NaCl, all strains were completely inactivated. These results indicate that YZ_2_ and YZ_4_ demonstrate strong osmotic pressure tolerance, whereas AC and YZ_3_ exhibit poor resistance to osmotic stress. LAB strains with high tolerance can stably survive and metabolize in complex and harsh production environments, providing solid support for large-scale industrial fermentation production. Thus, the good tolerance of XQ_1_, YZ_2_, and YZ_4_ provides conditions for future industrial production.

### 3.4. Antioxidant Activity of Strains

**Figure 6:**
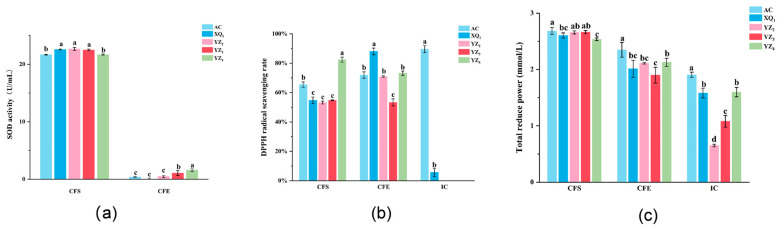
The antioxidant capacity of different strains was analyzed: SOD activity (**a**); DPPH radical scavenging ability (**b**); total antioxidant activity (**c**). Values are shown as means ± SD of three replications. CFS: cell-free supernatant; IC: intact cell; CFE: cell-free extract. Different letters in the same bar indicate significant differences (*p* < 0.05).

Antioxidant capacity pertains to the ability to neutralize or mitigate oxygen free radicals and other reactive species responsible for free radical formation. When assessing the quality of probiotics, the antioxidant potential of bacterial strains represents a fundamental criterion. The key parameters utilized for evaluating antioxidant capacity include SOD activity, total antioxidant capacity, and DPPH free radical scavenging ability. Among these, SOD activity serves a pivotal function in counteracting oxidative stress. Studies have indicated that LAB strains exhibiting elevated SOD expression may be leveraged for both conventional food applications and novel therapeutic interventions [26,37]. The results pertaining to SOD activity are depicted in Figure 6a. The CFS derived from all strains isolated from RRTP demonstrated substantial SOD activity, with the SOD activity of the CFS consistently surpassing that of the CFE across all strains. In contrast, no relevant activity was observed in the ICs (not presented in the figure). These findings align with those reported by Hongyu Wang et al. [19], suggesting a potential correlation with strain-specific metabolites, such as organic acids. Aksomtong Choonuk [38] reported that the CFE from 11 LAB strains isolated from the human oral cavity exhibited SOD activities ranging from 0 to 7 U/mL, demonstrating significant antioxidant potential—a finding consistent with our experimental observations. However, their study did not assess SOD activity in CFS. In contrast, our results revealed markedly higher SOD activity (>20 U/mL) across all CFS samples from the Rosa roxburghii-derived LAB strains, strongly suggesting exceptional SOD-related antioxidant capabilities.

The DPPH free radical scavenging capacity is illustrated in Figure 6b. The CFS and CFE obtained from the isolated strains XQ_1_, YZ_2_, YZ_3_, and YZ_4_ and the control strain AC demonstrated DPPH free radical scavenging rates exceeding 50%. LAB strains exhibiting DPPH free radical scavenging rates above 30% are generally regarded as possessing strong antioxidant activity [39], suggesting that the LAB isolated from RRTP exhibit notable antioxidant properties. Variations in DPPH free radical scavenging capacity were observed among the CFS, CFE, and ICs derived from the same strain. Within the ICs, strain AC displayed substantial DPPH free radical scavenging activity (>70%), whereas strain XQ_1_ exhibited minimal activity (<10%), and no activity was detected in strains YZ_2_, YZ_3_, and YZ_4_. However, contrary to most findings that indicate that ICs generally exhibit higher DPPH scavenging activity than LAB’s CFE [40], divergent results were obtained in this study, which may be attributed to distinct metabolic products. In the field of antioxidant research on LAB, ICs are regarded as non-enzymatic factors associated with antioxidant activity. Previous studies have reported that ICs contain substances such as glutathione, Mn^2+^, sulfhydryl groups (-SH), and peptides or amino acids, which exert positive synergistic effects on the antioxidant system of ICs, collectively maintaining intracellular redox homeostasis [41]. The CFS, on the other hand, is primarily linked to LAB metabolites, including organic acids, exopolysaccharides, and various enzymes [42,43]. Although progress has been made in LAB antioxidant research, their specific mechanisms remain to be fully elucidated [44]. Studies by Hongyu Wang et al. [19]. demonstrated that the DPPH radical scavenging rates of the LAB CFS and CFE exceeded those of ICs, with no detectable antioxidant activity in some ICs. Conversely, Li Kexin’s findings [40] indicated higher DPPH scavenging rates in LAB ICs compared to the CFS and CFE. A review by Tao Feng et al. [44]. highlighted discrepancies in DPPH scavenging rates between the CFS and ICs across studies, attributing these variations to differences in bacterial antioxidant systems, metabolite profiles, and strain-specific characteristics. In this study, the CFS of four *L. plantarum* strains (XQ_1_, YZ_2_, YZ_3_, and YZ_4_) isolated from *Rosa roxburghii* pomace exhibited superior DPPH scavenging rates compared to their ICs. Based on these results, it is hypothesized that these strains share similar antioxidant properties, with the enhanced DPPH scavenging capacity of the CFS likely attributable to metabolites such as organic acids, SOD, exopolysaccharides, and peroxidase (POD). Additionally, structural barriers posed by the cell walls and membranes of intact cells may hinder the interaction between intracellular antioxidants and DPPH radicals, thereby limiting the antioxidant performance of ICs.

The link between reducing power and antioxidant activity suggests that it can serve as a reliable indicator for assessing potential antioxidant capacity [40]. The total antioxidant activity, as measured by FRAP, is depicted in Figure 6c. In the CFS, the total antioxidant capacities of XQ_1_ (2.60 FeSO_4_·7H_2_O eq mmol/L), YZ_2_ (2.66 FeSO_4_·7H_2_O eq mmol/L), YZ_3_ (2.66 FeSO_4_·7H_2_O eq mmol/L), and YZ_4_ (2.54 FeSO_4_·7H_2_O eq mmol/L) were found to be comparable to that of the control strain AC (2.68 FeSO_4_·7H_2_O eq mmol/L). Hongyu Wang et al. [19] successfully isolated *Lactobacillus plantarum* and *Lactobacillus acidophilus* from traditional Chinese dairy products, both exhibiting high antioxidant capacity, with total antioxidant levels in the CFS recorded at 1.19 FeSO_4_·7H_2_O eq mmol/L and 1.21 FeSO_4_·7H_2_O eq mmol/L, respectively. In contrast, the LAB strains XQ_1_, YZ_2_, YZ_3_, and YZ_4_, which were isolated from RRTP in this study, exhibited considerably higher values in the CFS (>2.5 FeSO_4_·7H_2_O eq mmol/L), thereby highlighting the exceptional total antioxidant capacity of these strains. In the CFE, AC exhibited the highest reducing power. Within the ICs, although AC displayed markedly greater reducing power than the LAB strains isolated from RRTP, XQ_1_ (1.58 FeSO_4_·7H_2_O eq mmol/L), YZ_2_ (0.65 FeSO_4_·7H_2_O eq mmol/L), and YZ_4_ (1.60 FeSO_4_·7H_2_O eq mmol/L) still demonstrated remarkable total antioxidant capacity.

### 3.5. Safety Evaluation

#### 3.5.1. Antibiotic Susceptibility

Probiotic bacteria that exhibit antibiotic resistance have the potential to transfer corresponding antibiotic resistance genes to other intestinal microorganisms [45]. Consequently, evaluating the resistance profiles of probiotics is essential. Europe, recognized as one of the pioneers in probiotic research, has developed a well-established regulatory framework. In compliance with the EFSA guidelines for probiotic safety assessment, antibiotic susceptibility tests were performed on XQ_1_, YZ_2_, YZ_4_, and the commercially available control strain AC, which exhibited superior overall performance. The findings presented in Table 4 revealed that all LAB strains were susceptible to three widely used antibiotics: ampicillin, erythromycin, and tetracycline. Furthermore, AC displayed susceptibility to clindamycin and gentamicin, whereas YZ_2_ and YZ_4_ were sensitive to clindamycin. All four strains demonstrated resistance to kanamycin and chloramphenicol, while XQ_1_ exhibited resistance to clindamycin, and XQ_1_, YZ_2_, and YZ_4_ showed resistance to gentamicin. Prior studies have documented varying degrees of chloramphenicol resistance in certain LAB strains, such as *Lactobacillus johnsonii* [46]. Another investigation reported that *Lactobacillus reuteri* MG505 was susceptible to nine antibiotics, whereas LAB strains, including *Lactobacillus pentosus* 22B, *Lactobacillus plantarum* 21B, and *Enterococcus faecium* LC2V5, exhibited resistance to five antibiotics [47]. Additionally, research conducted by Horie et al. [48]. utilized MALDI-TOF to identify 450 high-scoring LAB strains from a pool of 1181 isolates, with the majority exhibiting resistance to clindamycin. *Lactobacillus plantarum* is known to possess intrinsic or natural resistance to certain antibiotics, with these resistance genes classified as non-transmissible [49]. It is important to note that LAB strains exhibiting resistance to clindamycin, gentamicin, kanamycin, and chloramphenicol are not necessarily hazardous, and further studies are required to determine the transmissibility of LAB resistance genes.

#### 3.5.2. Hemolysis Characteristics

**Figure 7:**
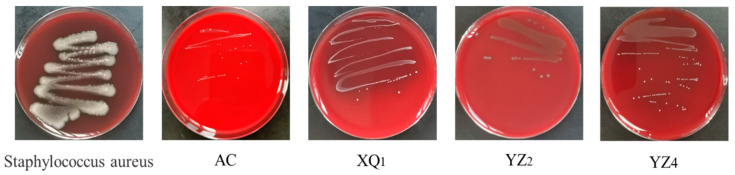
Hemolytic activity of strains was analyzed.

The evaluation of hemolytic activity is regarded as a key criterion in the selection of probiotic strains. Hemolysis refers to the rupture of red blood cells, resulting in the release of hemoglobin. The absence of a transparent zone is classified as non-hemolytic [50]. As illustrated in Figure 7, while *Staphylococcus aureus* exhibited pronounced hemolytic activity, the strains XQ_1_, YZ_2_, and YZ_4,_ which were isolated from RRTP, demonstrated either no hemolytic activity or only minimal hemolysis. These findings suggest that these three strains are safe and can provide a basis for further safety evaluations.

## 4. Conclusions

In this study, five acid-producing strains (XQ_1_, and YZ_1_–YZ_4_) were isolated from naturally fermented pomace of RRT in Guizhou’s karst region. A comparative evaluation of these isolates with the commercial strain AC was conducted to assess their probiotic attributes and ensiling viability, focusing on growth kinetics, acidification potential, tolerance to ethanol, acid, osmotic pressure, and bile salts, antimicrobial effects, antioxidant capacity, and safety profiles. The results showed that XQ_1_, YZ_2_, and YZ_4_ demonstrated robust acid production and growth capabilities, exhibited substantial environmental tolerance, and possessed notable antimicrobial and antioxidant properties. The three strains may serve as indigenous probiotic candidates for enhancing RRTP ensiling preservation or other prospective applications. In the future, full exploration of the potential of the strains for the horizontal gene transfer of antibiotic resistance genes, the molecular mechanism of strain-specific differences, and long-term stability is needed before commercial exploitation.

## Figures and Tables

**Table 1:** Surface characteristics of five suspected LAB strains.

Colony Morphology			Cell Morphology		
	Shape	Texture	Color	Morphology	Gram Staining
XQ_1_	Circular, raised colonies	Smooth and fine-textured	Milky white	Rod shaped	Gram-positive bacteria
YZ_1_	Circular, raised colonies	Thick and smooth	White with slight yellow	Rod shaped	Gram-positive bacteria
YZ_2_	Circular, raised colonies	Smooth and fine-textured	Milky white	Rod shaped	Gram-positive bacteria
YZ_3_	Irregular, serrated edges with depressions	Thick and wrinkled	White with slight yellow	Rod shaped	Gram-positive bacteria
YZ_4_	Circular, raised colonies	Smooth and fine-textured	Milky white	Rod shaped	Gram-positive bacteria

**Table 2:** Fermentation capability and physiological and biochemical characteristics of isolated strains.

	XQ_1_	YZ_1_	YZ_2_	YZ_3_	YZ_4_
Glucose	+	+	+	+	+
Fructose	+	+	+	+	+
Maltose	+	+	+	+	+
Lactose	+	−	+	+	+
Sucrose	+	+	−	+	+
Mannitol	−	+	+	−	−
Salicin	+	+	+	+	+
Cellobiose	+	+	+	+	+
Esculin	+	+	+	+	+
Sorbitol	+	−	+	+	+
Raffinose	−	−	+	−	+
Inulin	−	+	−	−	+
Produce acid	+	+	+	+	+
Glucose produce gas	−	−	−	+	−
Catalase	−	+	−	−	−
Motility	−	+	−	−	−
Starch Hydrolysis	−	−	−	−	−

Note: “+” indicates presence and “−” indicates absence.

**Table 3:** NCBI-BLAST.

Sample	Description	Total Score	Per. Ident	Accession
XQ_1_	*Lactobacillus plantarum* strain DMR17	2654	100.00%	MT229370.1
YZ_1_	*Bacillus licheniformis* strain BCRC 11702	2590	99.93%	NR116023.1
YZ_2_	*Lactiplantibacillus plantarum* strain HBUAS59638	2466	100.00%	ON125449.1
YZ_3_	*Weissella cibaria* strain II-I-59	2436	99.97%	NR036924.1
YZ_4_	*Lactiplantibacillus plantarum* strain JCM 1149	2521	99.93%	NR115605.1

**Table 4:** Antibiotic sensitivity of isolated strains to prescribed antibiotics.

		MIC (mg/L)			
Antibiotic	Breakpoint (mg/L)	AC	XQ_1_	YZ_2_	YZ_4_
Clindamycin	4	0.5	>128	1	4
Ampicillin	2	2	<0.125	<0.125	<0.125
Erythromycin	1	1	1	1	1
Tetracyclin	32	16	16	16	16
Gentamicin	16	8	>128	>128	>128
Kanamycin	64	>128	>128	>128	>128
Chloramphenicol	8	>128	>128	>128	>128
Vancomycin	n.r.	>128	>128	>128	>128
Streptomycin	n.r.	64	>128	>128	>128

Note: susceptible (green); resistant (Red); no relevant requirements (light blue); green (MIC < breakpoint); red (MIC > breakpoint).

## Data Availability

The original contributions presented in the study are included in the article, further inquiries can be directed to the corresponding author(s).
